# Synthesis, multifunctional properties, and photocatalysis of cobalt ferrite (CoFe₂O₄) nanoparticles

**DOI:** 10.1038/s41598-026-63309-1

**Published:** 2026-07-28

**Authors:** Reham K. Abd El Hamid, Fawzia I. Barakat, Roshdi Seoudi, Doaa A. Said

**Affiliations:** 1 Physics Department, Faculty of Women for Art, Sciences and Education, Ain Shams University, Cairo, Egypt; 2 Spectroscopy Department, Physics Research Institute, NRC, Dokki, 12622 Cairo Egypt

**Keywords:** Cobalt ferrite nanoparticles, Sol–gel synthesis, Structural, Magnetic, Dielectric properties, Photocatalytic performance, Chemistry, Materials science, Nanoscience and technology

## Abstract

This study focuses on synthesizing cobalt ferrite (CoFe₂O₄) nanoparticles via a sol-gel method using ethylene glycol (EG) and polyethylene glycol (PEG) as capping and stabilizing agents, and examines their effects on structural, optical, magnetic, dielectric, and photocatalytic properties. Various characterization techniques, including X-ray diffraction (XRD), Fourier-transform infrared spectroscopy (FTIR), transmission electron microscopy (TEM), and UV-Vis diffuse reflectance spectroscopy (DRS), were used to investigate the incorporation of CoFe₂O₄ and the stabilizers. Both EG- and PEG-capped nanoparticles exhibited a cubic spinel structure. PEG enhanced crystallinity and dispersion, whereas EG facilitated nucleation, yielding smaller, more agglomerated particles. Magnetic measurements revealed that PEG-capped nanoparticles had a higher saturation magnetization (70.1 emu·g⁻¹), whereas the EG-capped nanoparticles demonstrated greater coercivity (about 550 G). Dielectric studies indicated that PEG-derived samples exhibited higher permittivity and alternating current (AC) conductivity. In the photocatalytic degradation of Rhodamine B under UV light, EG-derived nanoparticles achieved 95.8% degradation in 115 min, while PEG-derived nanoparticles reached 96.2% degradation in 150 min. This study provides a clear comparative understanding of how the molecular weight and chain length of polyol stabilizers (EG and PEG) influence the structure-property relationships in CoFe₂O₄ nanoparticles.

## Introduction

Spinel ferrites have recently garnered significant attention, becoming the focus of extensive research in both fundamental and applied sciences, primarily due to their diverse and multifunctional properties. Recent advances in the study of spinel ferrites have demonstrated their exceptional stability, enhanced adsorption capabilities, and tunable optoelectronic characteristics, making them highly suitable for a wide range of applications. These materials show considerable promise in areas such as transformers, telecommunications, catalysis, sensors, microwave devices, high-density magnetic recording, and biomedical applications^1–3^. The superior magnetic properties of spinel ferrites contribute to improvements in energy efficiency, signal transmission, chemical catalysis, environmental sensing, wireless communications, data storage, and targeted drug delivery systems^4–6^. Structurally, spinel compounds possess a cubic crystal lattice conforming to the space group Fd3m, where metal cations occupy tetrahedral and octahedral coordination sites within a face-centered cubic arrangement of oxygen anions. Spinel ferrites are classified as normal, with the formula A^2+^B^3+^O_4_, in which A^2+^ cations occupy tetrahedral sites and B^3+^ cations occupy octahedral sites, and as inverse spinels, in which A^2+^ cations occupy octahedral sites while B^3+^ cations are distributed across both sites, significantly affecting their properties^7,8^. A notable example is cobalt ferrite (CoFe₂O₄), which adopts an inverse spinel configuration and exhibits favorable characteristics such as enhanced magnetomechanical coupling, significant magnetostriction, and cost-effectiveness, making it suitable for a variety of technological applications^9^. Ferrites can also be classified into soft and hard categories based on their magnetic properties. Soft ferrites have low coercivity, enabling easy magnetization and demagnetization, ideal for reversible applications. In contrast, hard ferrites exhibit high coercivity, allowing for sustained magnetization and making them suitable for permanent magnet applications^10^. CoFe₂O₄ is classified as a hard magnetic material due to its intermediate-to-high coercivity and moderate saturation magnetization, allowing for numerous technological applications, including magnetic resonance imaging (MRI) for diagnostic applications, magnetic hyperthermia in cancer therapies, catalytic processes for gas remediation and alkane oxidation, targeted drug delivery systems, biosensors for clinical diagnostics, and gas sensing devices^11^. The fabrication of cobalt ferrite (CoFe₂O₄) nanoparticles has employed various methodologies, including conventional ceramic processing^12^, co-precipitation^13^, hydrothermal synthesis^14^, thermal decomposition^15,16^, sol-gel techniques^17^, and combustion synthesis^18^. Despite the diversity of these approaches, a common limitation is the difficulty in controlling particle size and achieving monodispersity, both of which are crucial for ensuring reproducible performance in applications. Subsequent surface modifications of the synthesized nanoparticles have been conducted to enhance their functionality in specialized contexts, such as drug delivery systems, MRI contrast enhancement^19,20^, bioseparation protocols for the isolating proteins, DNA, and cells^21^, catalytic reactions^22^, ferrofluid formulations for thermal management^23^, high-density data storage^23^, and adsorption processes^24^. A notable advantage of CoFe₂O₄ nanoparticles is their enhanced magnetocrystalline anisotropy compared to other metal ferrite nanoparticles with similar saturation magnetization. This property results in a significantly reduced magnetic relaxation time, improving the efficacy of these nanoparticles in time-sensitive applications^25^. This investigation details a comprehensive chemical synthesis protocol for magnetic CoFe₂O₄ nanoparticles using ethylene glycol (EG) and polyethylene glycol (PEG) as stabilizing agents. These polymers provide colloidal stability and enhance nanoparticle dispersibility, thereby streamlining the synthetic process. Despite extensive research on CoFe₂O₄ nanoparticles, a systematic comparison of EG and PEG within a single sol-gel synthesis route has not been reported. To address this gap, we establish a unified framework that correlates EG and PEG with the structural, optical, magnetic, dielectric, and photocatalytic properties of CoFe₂O₄ nanoparticles. This study provides a comprehensive investigation of the physicochemical properties, synthesis strategies, and potential applications of cobalt ferrite (CoFe₂O₄) nanoparticles, with particular emphasis on the roles of (EG) and (PEG) as reducing, capping, and stabilizing agents. The findings elucidate the underlying structure–property performance relationships and provide practical guidelines for the rational design of CoFe₂O₄ nanomaterials for wastewater treatment and other advanced technological applications.

## Experimental

### Synthesis of CoFe₂O₄ nanoparticles

Cobalt ferrite (CoFe₂O₄) nanoparticles were synthesized using a sol–gel technique. A precursor solution was prepared by dissolving 3 g of ferric chloride (FeCl_3_) and 1.5 g of cobalt chloride (CoCl₂) in 150mL of deionized water. Continuous magnetic stirring was employed to ensure thorough homogenization of the solution. To facilitate the formation of a gel network and to regulate the nucleation and growth of nanoparticles, 28mL of ethylene glycol (C₂H₆O₂) (EG) was used as a polyol medium. Citric acid (C_6_H_8_O_7_) was added as a chelating agent to enhance metal-ion coordination, thereby promoting the formation of a uniform and stable gel matrix. The mixture was stirred and maintained at 70 °C for 30 h to facilitate gelation The resultant gel-like precipitate was dried at 200 °C for 24 h to eliminate residual solvents and moisture. Following the drying process, the material was manually milled to yield a fine precursor powder. This powder was then calcined at 750 °C for 4 h in a muffle furnace, resulting in phase-pure crystalline CoFe₂O₄ nanoparticles. The 4-hour duration was selected to achieve complete crystallization and grain growth without excessive particle sintering. A parallel synthesis was conducted using polyethylene glycol (PEG) as the polyol medium, allowing for a comparative analysis of the structural, optical, and magnetic properties of the nanoparticles produced from both EG and PEG.

### Characterization techniques

The X-ray diffraction (XRD) analysis was conducted using a Philips X’Pert Pro diffractometer (Billerica, MA, USA), equipped with Cu Kα radiation (λ = 1.5406 Å). The instrument was operated at 45 kV and 40 mA, covering a 2θ° range from 5° to 80°. This analytical technique was used to determine the crystal structure of CoFe₂O₄, assess phase purity, and estimate crystallite size. To examine particle morphology and size distribution, transmission electron microscopy (TEM) was performed with a JEOL JSM100 CX microscope (Shimadzu, Japan) at an accelerating voltage of 80 kV. Additionally, complementary vibrational analysis was carried out using Fourier-transform infrared spectroscopy (FTIR) with a Bruker VERTEX 80 spectrometer (Optics Inc., Billerica, MA, USA) to identify functional groups and confirm key structural features. Diffuse reflectance UV-Vis spectra were obtained with a Jasco V-670 double-beam spectrophotometer (AzoNetwork, UK), spanning a wavelength range of 250 to 2500 nm and achieving a spectral resolution of 2 nm. The bandgap energy of the samples was determined through Tauc plot analysis. Magnetic properties were assessed using a vibrating sample magnetometer (VSM, Lake Shore 7410) under an applied magnetic field of 20kOe at room temperature. This focused on parameters such as saturation magnetization and coercivity to characterize the magnetic behavior of CoFe₂O₄. Finally, the dielectric properties were measured at room temperature using a Novocontrol Concept 40 broadband dielectric spectrometer (Montabaur, Germany) across a frequency spectrum ranging from 0.1 Hz to 20 MHz. To ensure accurate electrical characterization, both sides of each sample were coated with a thin layer of graphite, which served as electrodes.

### Catalytic activity of cobalt ferrite (CoFe₂O₄) nanoparticles

The photocatalytic activity of cobalt ferrite (CoFe₂O₄) nanoparticles, synthesized via (EG) and (PEG) methods, was evaluated by the degradation of Rhodamine B (RhB) under ultraviolet (UV) irradiation (λ = 365 nm) at ambient temperature (25 ± 1 °C). The experimental were conducted in a cylindrical glass reactor using 50 mg of catalyst dosage suspended in 100mL of a 10 mg/L RhB solution. The reaction mixture was stirred in the dark for 50 min to establish an adsorption-desorption equilibrium, after which it was irradiated with a 125 W high-pressure mercury lamp positioned 15 cm from the reactor surface. Continuous magnetic stirring was maintained throughout the irradiation to ensure a uniform exposure. Sampling for analysis was performed at predetermined intervals, with 5mL aliquots withdrawn every 15 min, up to 115 min for the EG-synthesized nanoparticles and 155 min for the PEG-synthesized ones. After sampling, the catalyst was separated from the solution through centrifugation at 5000 rpm for 10 min. The residual concentration of RhB in the supernatant was quantified by measuring the absorbance at the maximum wavelength $({\lambda}_{max}=554nm)$ using a Jasco V-670 double-beam UV-Vis spectrophotometer. The experiments were systematically replicated in triplicate for both nanoparticle synthesis methods to enable a thorough comparison of their photocatalytic efficiencies and to ensure the reliability and reproducibility of the results.

## Results and discussion

### XRD of CoFe₂O₄ nanoparticles

**Fig. 1:**
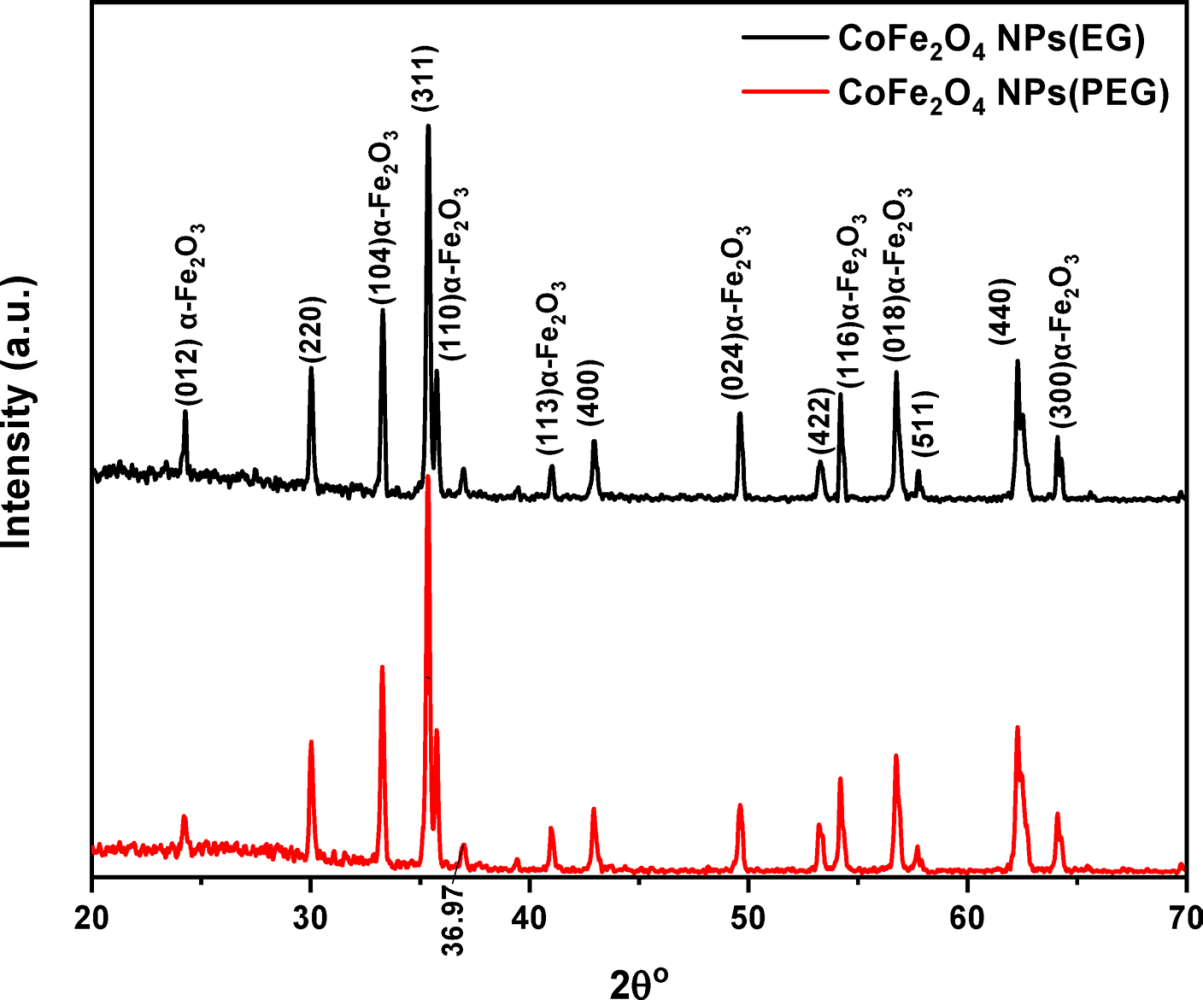
Description of the XRD pattern of CoFe_2_O_4_ nanoparticles synthesized using (EG) and (PEG) as reducing and capping agents.

X-ray diffraction (XRD) analysis was performed on CoFe₂O₄ nanoparticles capped with (EG) and (PEG), as illustrated in Fig. 1. The resulting diffraction patterns confirm the successful synthesis of a cubic spinel structure (space group Fd-3 m), evidenced by characteristic peaks located at 2θ values of approximately 30.03°, 35.37°, 42.93°, 53.27°, 57.75°, and 62.29°. These peaks correspond to the (220), (311), (400), (422)/(511), and (440) crystalline planes, aligning closely with the data from JCPDS card No. 22-1086. In addition to the prominent peaks, several weaker reflections were observed at (2q^o^) 24.27° (012), 33.29° (104), 35.75° (222), 41.03° (113), 49.61° (313), 54.19° (116), 57.75° (018), and 64.11° (300). These reflections suggest the presence of a minor hexagonal hematite (α-Fe₂O₃) phase, according to JCPDS card No. 33–0664^26–28^. A semi-quantitative phase analysis using the Reference Intensity Ratio (RIR) method quantified the hematite impurity in CoFe₂O₄. The weight percentages were derived from the integrated intensities of the (311) plane for CoFe₂O₄ and the (104) plane for α-Fe₂O₃, using RIR values of 1.60 and 2.40, respectively. The EG-capped sample contained about 23.9 wt% hematite, while the PEG-capped sample had 25.1 wt%. This 1.2 wt% difference is within the experimental uncertainty (± 2–3%), indicating that replacing EG with PEG as a capping agent does not significantly impact phase purity or spinel ferrite formation, as both methods yield a cubic spinel structure with minor hematite impurity. Notably, the XRD pattern of the PEG-capped sample exhibits sharper and more intense peaks, particularly at the (311) reflection, compared to the EG-capped specimen. This observation indicates that PEG enhances crystallographic growth and facilitates the formation of larger ordered crystallographic domains, while the smaller EG molecules promote rapid nucleation, resulting in less ordered and more defective crystallites^29,30^. The crystallite size was determined from the most intense (311) peak using the Scherrer equation^31^:

D = kλ / (β cos θ).

where k = 0.9, λ = 1.5406 Å (Cu Kα radiation), β is the full width at half maximum (in radians), and θ is the Bragg angle. The average crystallite sizes calculated were 27.86 nm for the EG-capped CoFe₂O₄ nanoparticles and 29.85 nm for those capped with PEG. The enhanced crystallinity observed with PEG (crystallite size: 29.85 nm vs. 27.86 nm for EG) is attributed to PEG’s longer polymeric chains providing superior steric stabilization, promoting ordered growth. Conversely, the smaller EG molecules facilitate rapid nucleation, resulting in smaller, more defective crystallites^29,32^.

### TEM analysis of CoFe₂O₄ nanoparticles

**Fig. 2:**
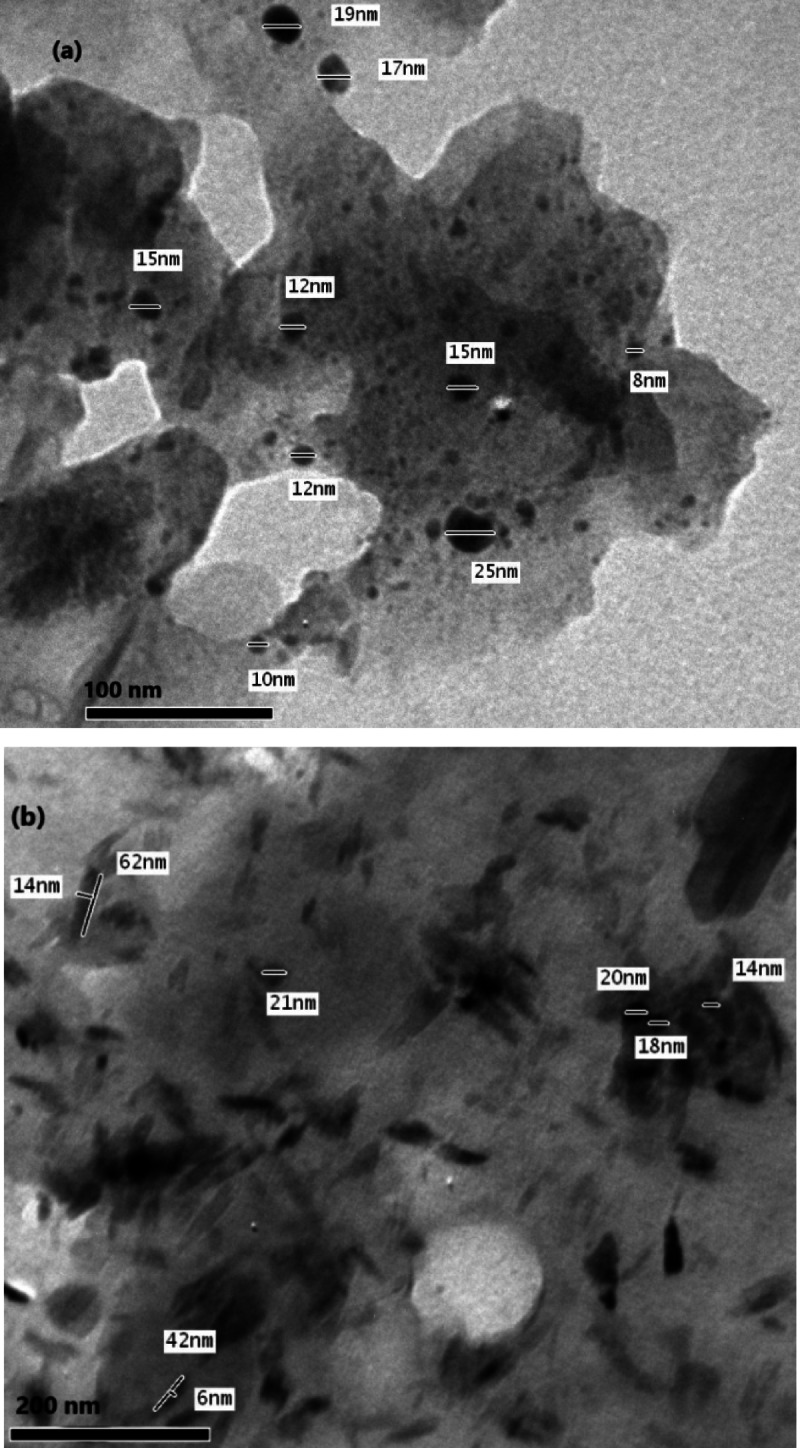
Transmission Electron Microscope images of CoFe₂O₄ nanoparticles synthesized using (**a**) (EG) and (**b**) (PEG) as reducing and capping agents.

Transmission Electron Microscopy (TEM) was utilized to investigate the morphology and size distribution of CoFe₂O₄ nanoparticles synthesized through the sol-gel technique, employing (EG) and (PEG) as reducing and capping agents. The TEM images, illustrated in Figs. 2 (a) and 6(b), reveal significant differences in the structural characteristics of the synthesized nanoparticles. The CoFe₂O₄ nanoparticles produced using EG, as shown in Fig. 2 (a), exhibit irregular morphology and significant agglomeration. The particle diameters range from 8 to 25 nm, with an average size of approximately 19.3 nm. The pronounced clustering observed in these nanoparticles suggests that, although EG is an effective reducing agent, its short-chain structure is insufficient for adequate steric stabilization. This leads to uncontrolled particle growth and coalescence, which is consistent with findings reported in the literature^29,30^. Conversely, the nanoparticles synthesized with PEG, depicted in Fig. 2 (b), demonstrate significantly improved dispersion and reduced agglomeration. The particle sizes of these PEG-assisted nanoparticles range from 6 to 62 nm, yielding an average diameter of approximately 27.4 nm. The enhanced dispersity and diminished agglomeration can be attributed to the longer polymeric chains of PEG, which provide substantial steric hindrance and superior surface passivation. The broader size distribution observed for the PEG-derived nanoparticles indicates an alteration in nucleation and growth kinetics associated with PEG’s dual role as both a reducing and capping agent^29,33^. These contrasting trends underscore the formation of smaller, heavily agglomerated particles in the presence of EG, as opposed to larger, well-dispersed particles when PEG is employed. This behavior aligns with previous studies on polyol-mediated ferrite synthesis^4,29^. Furthermore, these observations correlate well with crystallite sizes obtained from X-ray Diffraction (XRD) analyses. Overall, these findings emphasize the significant influence of capping agent molecular weight on the morphology and colloidal stability of nanoparticles, providing a clear approach to tailoring CoFe₂O₄ nanostructures for various applications in magnetic sensors, hyperthermia, and catalysis^4^.

### FTIR analysis of CoFe₂O₄ nanoparticles

**Fig. 3:**
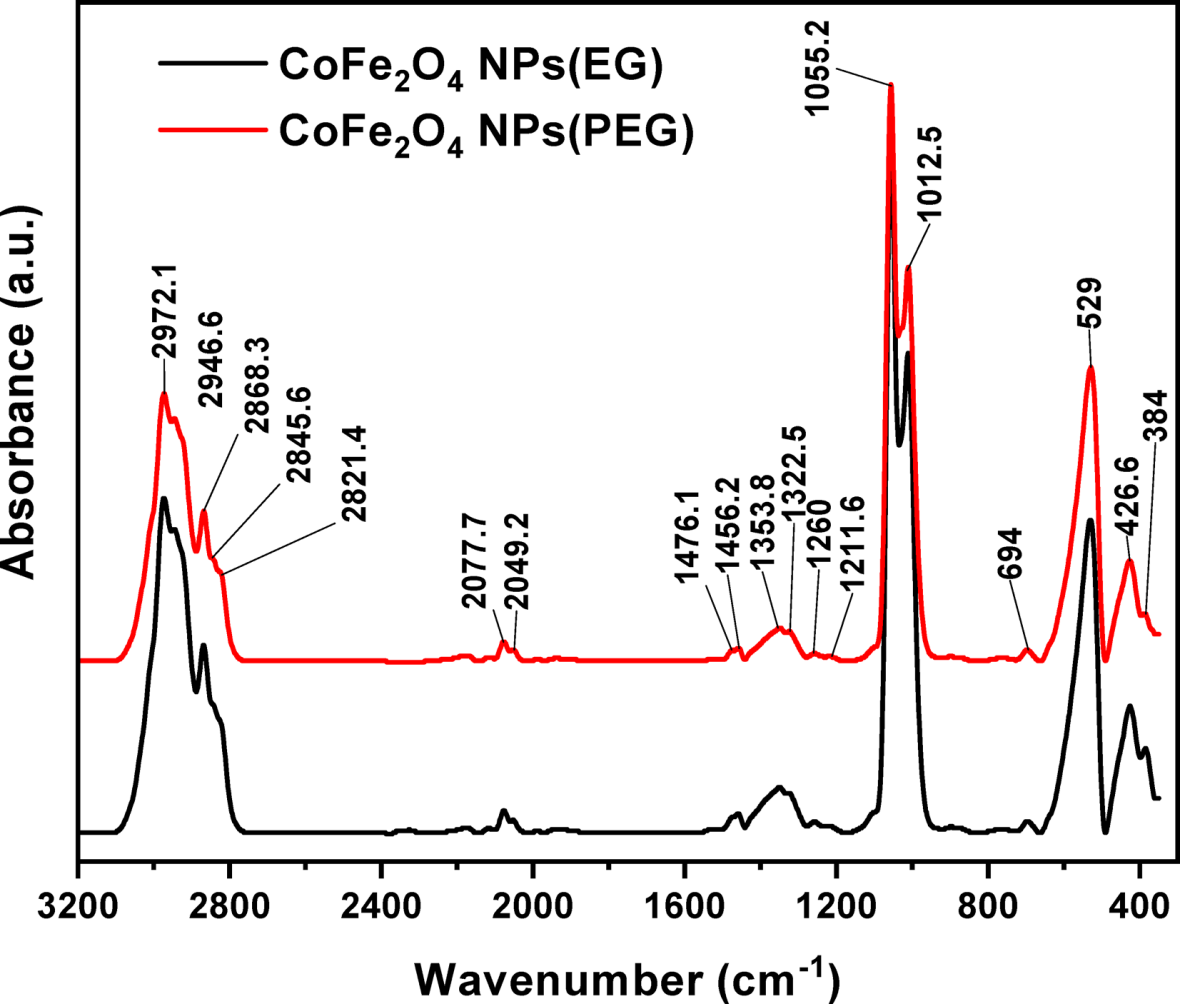
Description of the FTIR Spectroscopy of CoFe_2_O_4_ nanoparticles synthesized using (EG) and (PEG) as reducing and capping agents.

The functional groups and structural characteristics of CoFe₂O₄ nanoparticles synthesized using (EG) and (PEG) were analysed via FTIR spectroscopy in the spectral range of 4000–400 cm⁻¹. The FTIR spectra for both samples (Fig. 3) reveal significant similarities, indicating the successful formation of the spinel ferrite phase, corroborated by the surface capping from the polyol solvents. A broad band at 2912 cm⁻¹ is attributed to the O–H stretching vibration, associated with adsorbed water or residual hydroxyl groups from the solvents. A weaker bandat 1353 cm⁻¹ corresponds to the O–H bending modes, further confirming the presence of hydroxyl species^34,35^. The symmetric C–H stretching vibration appears at 2868 cm⁻¹, indicating that alkyl chains from both EG and PEG remain on the surface of the nanoparticles. A characteristic band at 1055 cm⁻¹ is attributed to the C–O ether stretching, suggesting effective capping with both EG and PEG^36,37^.

A band near 1476 cm⁻¹, associated with C = O stretching, likely results from partial oxidation of the polyol. The weak bands at 1456 cm⁻¹ and 1021 cm⁻¹ are linked to the asymmetric stretching and bending vibrations of organic compounds or the presence of polyol-related functional groups (C–H, C–O, O–H). Also, these may come from the synthesis environment or residual metal chlorides, which were mostly removed during washing and heat treatment^38,39^. In the lower wavenumber region, distinct metal–oxygen (M–O) vibrational bands at approximately 694, 529, and 429 cm⁻¹ are characteristic of the spinel ferrite structure^40^. The band at 694 cm⁻¹ corresponds to Fe³⁺–O²⁻ stretching at tetrahedral (A) sites, while the 529 cm⁻¹ band pertains to octahedral (B) site stretching of Fe³⁺–O²⁻ or Co²⁺–O²⁻^41,42^. The lower-frequency band at 429 cm⁻¹ corresponds to Co²⁺–O²⁻ octahedral or mixed with Fe³⁺–O²⁻ bonds^42^. Variations in band positions and intensities observed between the EG- and PEG-capped samples may result from differences in cation distribution andbond lengths, influenced by particle size and interactions with the capping agents^43^. The presence of polyol-related functional groups (C–H, C–O, O–H) confirms effective surface passivation, enhancing colloidal stability and reducing agglomeration. The consistent M–O vibrational bands across samples validate the formation of the inverse spinel structure of CoFe₂O₄, characterized by Fe³⁺ ions predominantly occupying the tetrahedral sites and a mixed distribution of Fe³⁺ and Co²⁺ in the octahedral sites. The fundamental relationship among vibrational frequency, atomic masses, and bond strength can be described by Hooke’s Law in the context of a diatomic harmonic oscillator, expressed mathematically as $\:\left(\nu=\frac{1}{2\pi\:c}\sqrt{\frac{k}{\mu\:}}\right)$, where v represents the vibrational wavenumber, c is the speed of light, k indicates the force constant, and $\:\mu\:$ is the reduced mass of the two atoms in the diatomic molecule. For the iron-oxygen bond (Fe–O), the force constant is approximately (k ~ 210.3 N/m)^44^, while for the cobalt-oxygen bond (Co–O), it is about ( k ~ 130.8 N/m)^45^. Based on these values, the wavenumber for the tetrahedral Fe–O bond is calculated to be around (535.7 cm^− 1^), while the wavenumber for the octahedral Co–O bond is approximately (420.1 cm^− 1^). These theoretical predictions are in good agreement with the corresponding experimental observations, highlighting the effectiveness of the harmonic oscillator model in describing vibrational behavior in these complexes.

### UV-visible diffuse reflectance spectra of CoFe₂O₄ nanoparticles

**Fig. 4:**
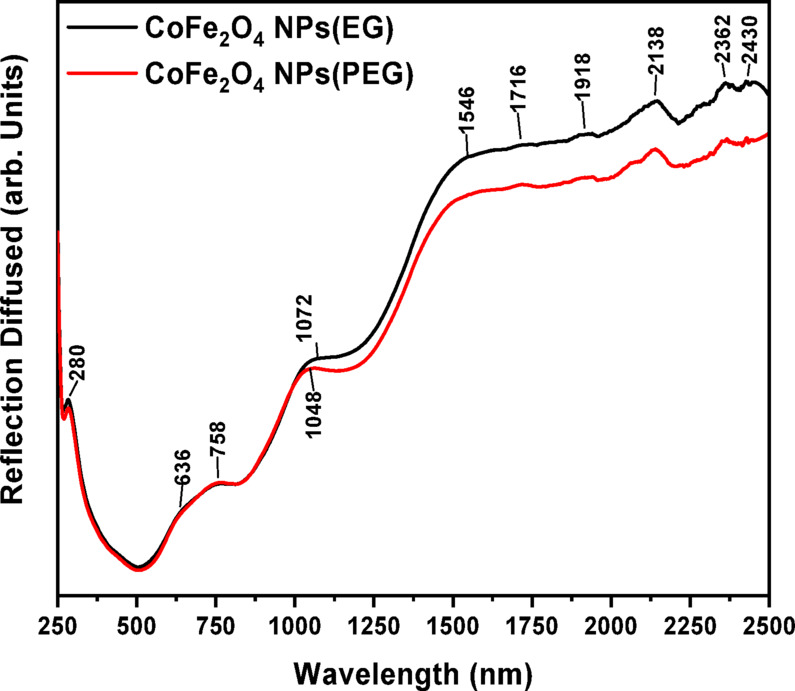
Reflection diffused spectra of CoFe_2_O_4_ nanoparticles synthesized using (EG) and (PEG) as reducing and capping agents.

**Fig. 5:**
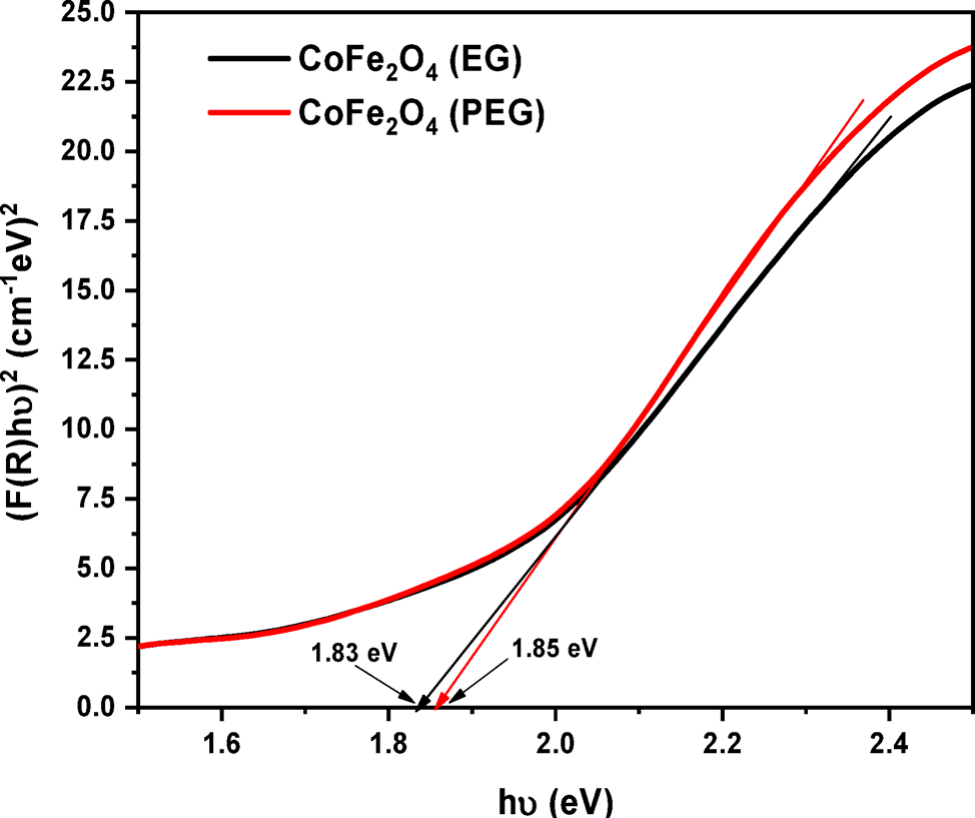
Description of the direct band gap of CoFe_2_O_4_ nanoparticles synthesized using (EG) and (PEG) as reducing and capping agents.

**Fig. 6:**
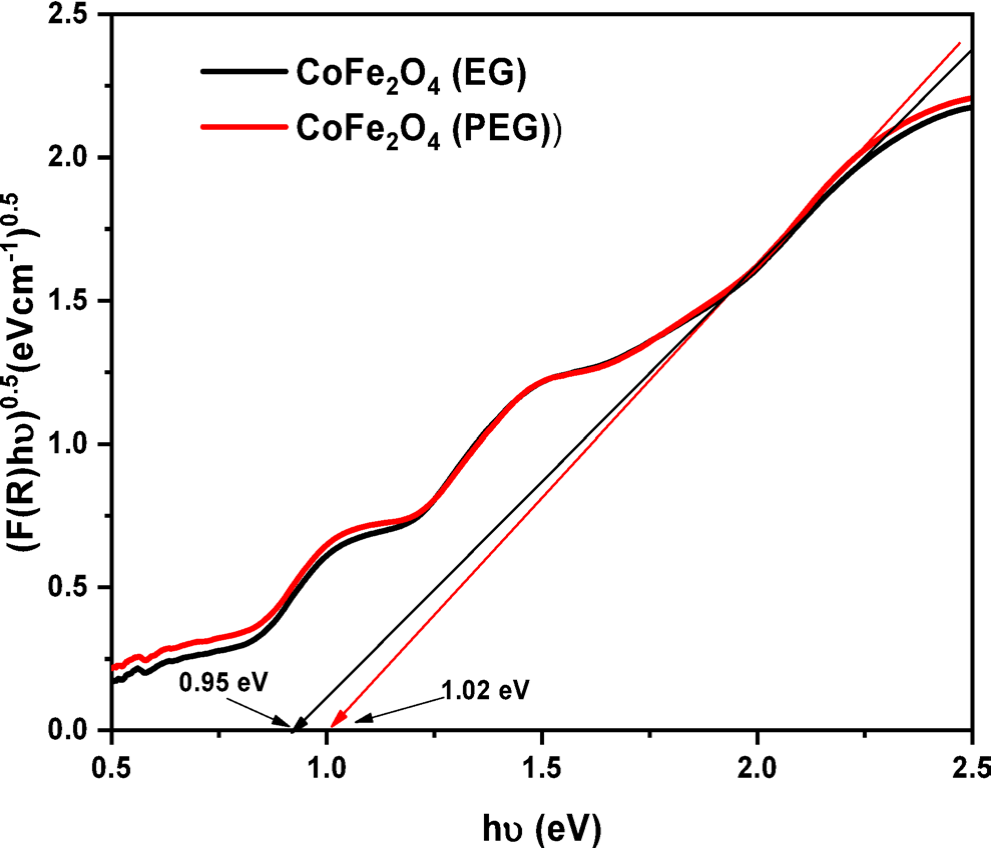
Description of the indirect band gap of CoFe_2_O_4_ nanoparticles synthesized using (EG) and (PEG) as reducing and capping agents.

The optical properties, including the band gap characteristics, of CoFe₂O₄ nanoparticles synthesized using (EG) and (PEG) were systematically examined via UV–vis diffuse reflectance spectroscopy (DRS), as shown in Fig. 4. The diffuse reflectance spectra show distinct bands characteristic of d-d transitions in tetrahedrally coordinated Co²⁺ ions in the spinel lattice. The 3d orbital levels of Co²⁺ are split in the tetrahedral crystal field created by nearby O²⁻ ions, facilitating these transitions. A prominent peak at 280 nm indicates an intrinsic band-to-band transition. Notably, a redshift occurs from 1048 nm (in PEG) to 1072 nm (in EG). This shift is attributed to the smaller crystallite size of the EG-derived sample, which measures 27.86 nm according to XRD analysis, compared to the 29.85 nm of the PEG-derived sample. The smaller crystallite size leads to increased quantum confinement effects and changes in the electronic structure^29,32^. Additionally, the observed redshift indicates thermal effects during synthesis that affect the structural ordering and cation distribution of the CoFe₂O₄ ferrite nanoparticles. The optical band gap (Eg) was deduced utilizing the Tauc relation^46,47^:

$$\:{\left(\alpha\:h\upsilon\right)}^{n}=A\left(h\upsilon-{E}_{g}\right)$$

where $\:\alpha\:$ is the absorption coefficient, $\:A$ is a constant, $\:h\upsilon$ is the photon energy, and $\:n$ denotes the transition index (with 1/2 for indirect transitions and 2 for direct transitions), and $\:{E}_{g}$ refers to the band gap energy^32^. The absorption coefficient α is associated with the Kubelka–Munk function $\:F\left({R}_{d}\right)$ through the relation^32,48^:

$$\:F\left({R}_{d}\right)=\frac{{(1-{R}_{d})}^{2}}{2\:{R}_{d}}$$

where R represents the measured diffuse reflectance^48^. The characterization of direct versus indirect bandgap transitions in CoFe₂O₄ is fundamentally rooted in its inverse spinel electronic structure. In CoFe₂O₄, the valence band maximum is predominantly derived from the strong hybridization of O 2p and Co 3d orbitals, while the conduction band minimum is primarily associated with Fe 3d states located at tetrahedral sites. The optical transitions in this material are primarily direct in nature, owing to the momentum-conserving transitions between the O 2p/Co 3d hybridized states and the Fe 3d states occurring at the same k-point in the Brillouin zone. However, factors such as structural disorder, cation mixing, and defects within the spinel lattice can lead to the emergence of phonon-assisted indirect transitions, particularly at lower energy levels. Consequently, both direct (*n* = 2) and indirect (*n* = 1/2) transitions were investigated in this study. The Tauc plots demonstrate enhanced linearity for the direct transition model, which aligns with the predominant direct bandgap character of CoFe₂O₄ spinel ferrites, as supported by previous research^32,49^. The direct band gap was calculated by plotting $\:{\left[F\left({R}_{d}\right)h{\upnu\:}\right]}^{2}$ against $\:h\upsilon$ (eV) (see Fig. 5). The extrapolation of the linear region to the energy axis yielded direct band gap values of 1.83 eV for CoFe₂O₄ synthesized in EG and 1.85 eV for CoFe₂O₄ synthesized in PEG. The indirect band gap was evaluated from the plot of $\:{\left[F\left({R}_{d}\right)h{\upnu\:}\right]}^{1/2}\:$versus $\:h\upsilon$ (eV) (refer to Fig. 6), resulting in values of 0.95 eV for CoFe₂O₄ (EG) and 1.02 eV for CoFe₂O₄ (PEG). The observed variation in the indirect band gap across the samples may reflect alterations in carrier concentration and lattice disorder. The comparatively lower indirect band gap suggests an increased carrier concentration and enhanced sub-band transitions at shorter wavelengths, while the band gap tends to increase with longer wavelengths due to diminishing effects of absorption tailing^49^. Our band gap values (1.83–1.85 eV direct, 0.95–1.02 eV indirect) align with recent reports for CoFe₂O₄ nanoparticles synthesized via solvothermal methods^50^.

### Magnetic properties of CoFe₂O₄ nanoparticles

**Fig. 7:**
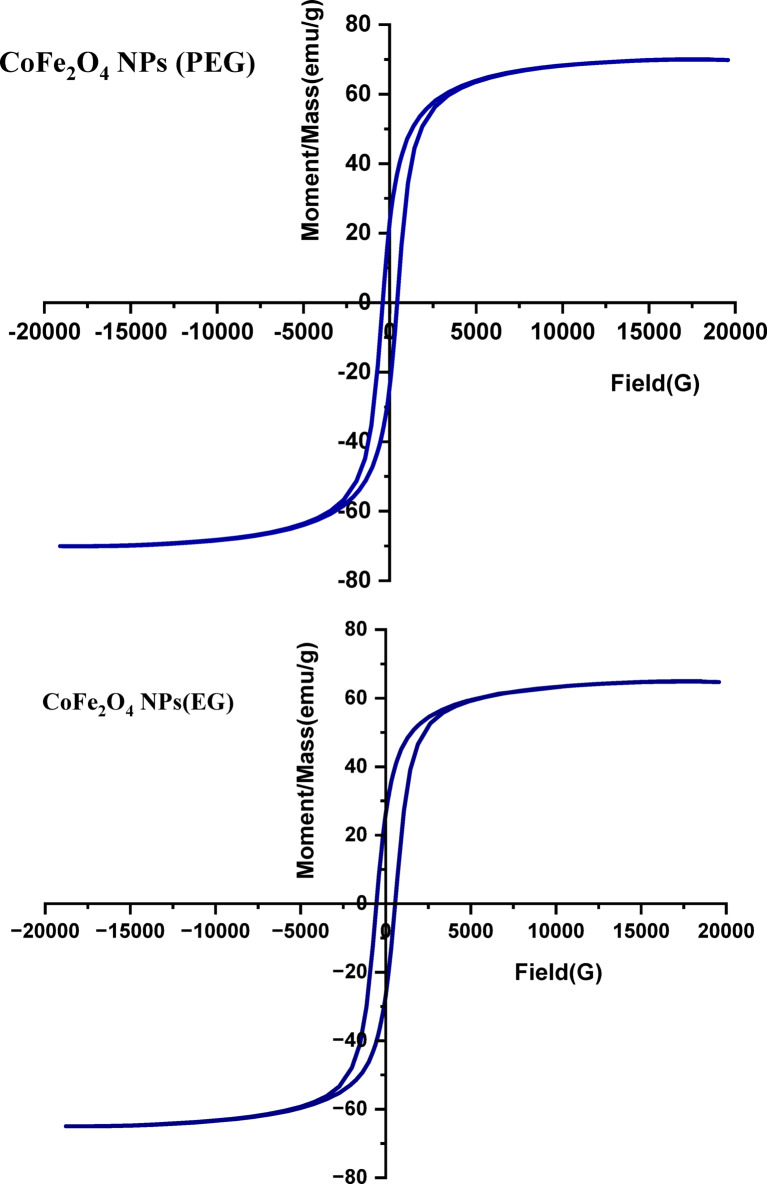
Description of the room-temperature magnetic hysteresis loops (M–H curves) of CoFe₂O₄ nanoparticles synthesized using EG and PEG as reducing and capping agents.

Figure 7 (a, b) illustrates the magnetic hysteresis loops of cobalt ferrite (CoFe₂O₄) nanoparticles synthesized using the EG and PEG methods. Both nanoparticle samples exhibit characteristic ferrimagnetic behavior at room temperature, with key parameters including saturation magnetization (Ms), remanent magnetization (Mr), and coercivity (Hci) summarized in Table 1. Notably, CoFe₂O₄ nanoparticles synthesized with PEG display a higher saturation magnetization (Ms ≈ 70.1 emu·g⁻¹) than those synthesized with EG (Ms ≈ 65.0 emu·g⁻¹). This increase in Ms for nanoparticles synthesized using PEG can be attributed toenhanced crystallinity and reduced surface spin canting. This improvement is a direct result of PEG’s effective surface passivation, which minimizes surface defects. Additionally, there are stronger superexchange interactions between Co²⁺ and Fe³⁺ ions, also resulting from the effective surface passivation provided by longer PEG chains^4,29,31^. In contrast, nanoparticles synthesized via the EG route exhibit significantly higher coercivity (Hci ≈ 547.9 Oe) and remanent magnetization (Mr ≈ 26.317 emu·g⁻¹) compared to their PEG-based counterparts (Hci ≈ 417.9 Oe; Mr ≈ 23.576 emu·g⁻¹). The higher coercivity (Hci ≈ 547.9 Oe) observed in EG samples, which have a smaller crystallite size of 27.86 nm, compared to PEG-derived samples with a crystallite size of 29.85 nm, can be attributed to a greater surface-to-volume ratio. This increased ratio results in a higher surface spin disorder. Furthermore, the presence of antiferromagnetic α-Fe₂O₃ on the surfaces of the particles contributes to exchange bias interactions, which enhance coercivity, particularly in EG-derived samples. The distribution of Fe³⁺ and Co²⁺ ions between tetrahedral and octahedral sites also plays a critical role in determining the magnetocrystalline anisotropy constant, which influences coercivity. In contrast, the enhanced crystallinity in PEG samples may lead to a more ordered distribution of cations, resulting in reduced anisotropy fluctuations and consequently lower coercivity^43^. Consequently, these EG-derived particles possess a greater squareness ratio (Mr/Ms ≈ 0.407 vs. 0.338) and a larger hysteresis loop area (1.124 × 10⁵ erg·g⁻¹ vs. 9.279 × 10⁴ erg·g⁻¹). The increased coercivity and hysteresis loss in the EG-synthesized sample are attributed to smaller particle size, higher lattice strain, and enhanced surface anisotropy, which collectively hinder domain-wall motion and elevate magnetic anisotropy. The comparatively softer magnetic behavior of PEG-synthesized CoFe₂O₄ nanoparticles (characterized by lower Hci and a narrower hysteresis loop) results from reduced interparticle interactions and improved magnetic homogeneity, both due to the superior steric stabilization provided by PEG. These findings highlight the importance of selecting the appropriate polyol to precisely tune magnetic properties: the EG synthesis route produces harder magnetic nanoparticles suitable for permanent magnets and magnetic recording, while the PEG route yields nanoparticles with higher Ms and softer magnetic behavior, making them ideal for biomedical applications, magnetic hyperthermia, and high-frequency devices and cytotoxicity^51–56^. Additionally, the observed dependence of magnetization on particle size and crystallinity is consistent with results from transmission electron microscopy (TEM) and X-ray diffraction analyses.

**Table 1:** Magnetic parameters extracted from the hysteresis loops of CoFe₂O₄ nanoparticles synthesized using (EG) and (PEG).

Magnetic Parameters	CoFe₂O₄ (EG)	CoFe₂O₄ (PEG)
Total hysteresis loop Area	$\:1.1244\times\:\:{10}^{5}\:$erg·g⁻¹	$\:\:9.279\times\:{10}^{4}\:$erg·g⁻¹
Intrinsic coercivity (Hci)	547.90 Oe	417.90 Oe
Loop flatness parameter	0.70795	0.72027
Negative intrinsic coercivity (Hci)	-545.27 Oe	-415.56 Oe
Positive Intrinsic Coercivity (Hci)	550.52 Oe	420.34 Oe
Saturation magnetization (Ms)	65.0 emu·g⁻¹	70.1 emu·g⁻¹
Negative remanent magnetization (Mr)	-26.616 emu·g⁻¹	-23.748 emu·g⁻¹
Positive remanent magnetization (Mr)	26.317 emu·g⁻¹	23.576 emu·g⁻¹
Negative saturation magnetization (Ms)	-64.986 emu·g⁻¹	-70.076 emu·g⁻¹

### Frequency-dependent dielectric and conductivity properties (1 Hz – 20 MHz)

**Fig. 8:**
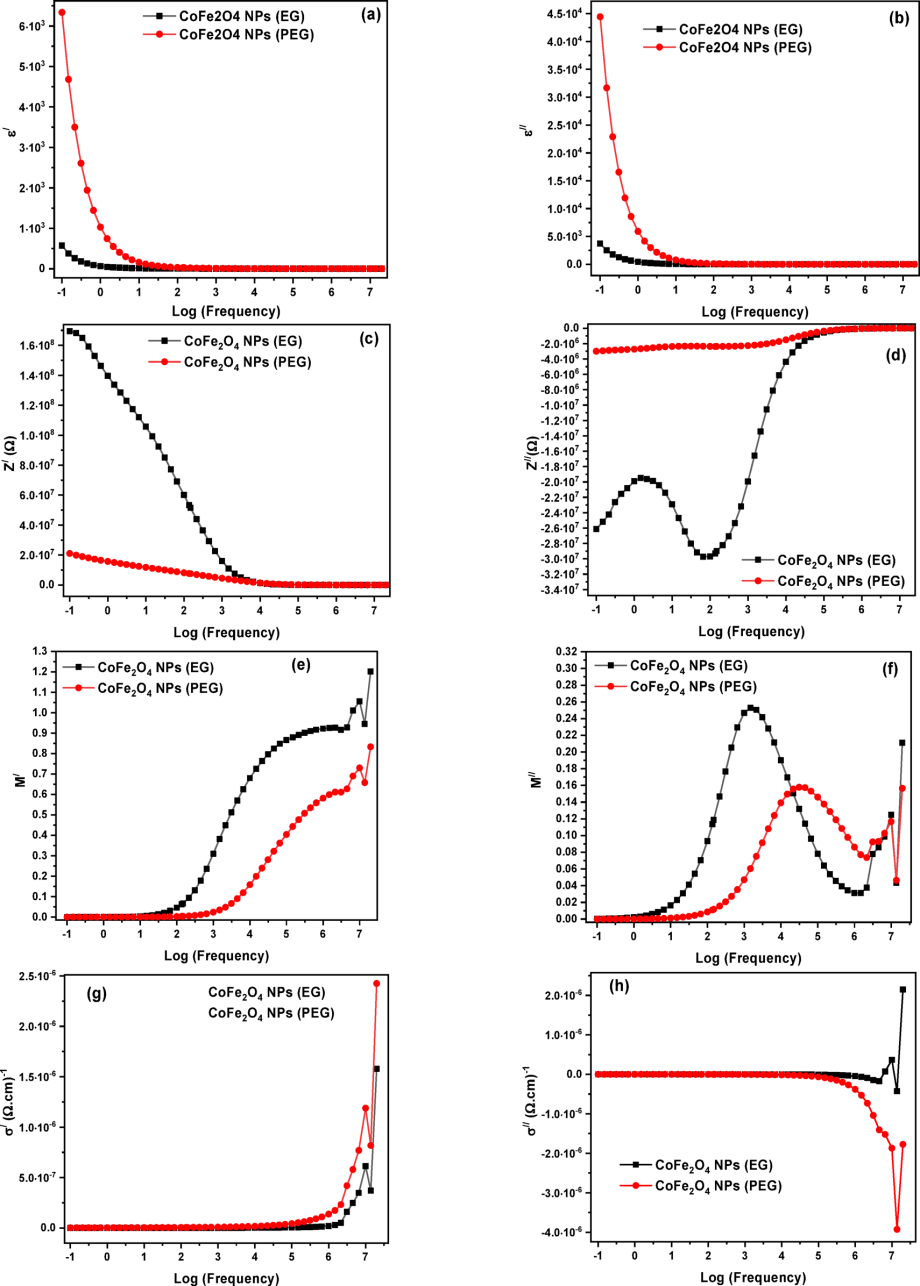
Frequency-dependent dielectric properties of CoFe₂O₄ ferrite nanoparticles capped with (EG, black lines) and (PEG, red dots). (**a**) Real part of the dielectric permittivity (ε′) versus log(frequency). (**b**) Imaginary part of the dielectric permittivity (ε″) versus log(frequency). (**c**) Real part of the impedance (z′) versus log(frequency). (**d**) Imaginary part of the impedance (z′′) versus log(frequency). (**e**) Real part of the electric modulus (M′) versus log(frequency). (**f**) Imaginary part of the electric modulus (M″) versus log(frequency). (**g**) Real part of the electric conductivity (s′) versus log(frequency). (**h**) Imaginary part of the electric conductivity (σ″ ) versus log(frequency).

^62,63^. The proposed photocatalytic degradation mechanism is illustrated schematically in Fig. 10. CoFe₂O₄ is classified as a narrow-band-gap semiconductor, with a calculated energy band gap (Eg) of approximately 1.83–1.85 eV, derived from UV-Vis spectra^64,65^. Upon exposure to light irradiation, electrons are excited from the valence band (VB) to the conduction band (CB), leading to the generation of electron-hole pairs (e⁻/h⁺). The photogenerated electrons actively reduce dissolved O₂ to form superoxide radicals (•O₂⁻), while the corresponding holes oxidize surface-adsorbed H₂O or OH⁻, generating hydroxyl radicals (•OH)^63^. XRD analysis identified a minor α-Fe₂O₃ phase in CoFe₂O₄ samples, suggesting the formation of a CoFe₂O₄/α-Fe₂O₃ heterostructure. This close contact may enhance charge separation and the generation of reactive oxygen species, thereby improving RhB degradation. The presence of α-Fe₂O₃ creates an interfacial heterojunction that promotes the transfer of photogenerated charge carriers, reduces electron–hole recombination, and boosts reactive oxygen species generation, which is crucial for effective RhB degradation. For RhB degradation, both •OH and •O₂⁻ radicals interact with the conjugated xanthene chromophore, resulting in stepwise N-deethylation and chromophore cleavage. This cascade of reactions culminates in rapid discoloration and mineralization of the dye^65,66^. The reactive oxygen species (ROS) generated play a pivotal role in the effective degradation of pollutants.

The dielectric constant can be quantitatively represented as ($\:\epsilon\:={\epsilon\:}^{{\prime\:}}-j{\epsilon\:}^{{\prime\:}{\prime\:}}$), where the real part ($\:{\epsilon\:}^{{\prime\:}}$) denotes the stored energy, while the imaginary part (ε″) signifies dissipated energy. The dielectric characteristics of spinel ferrites are significantly influenced by structural homogeneity, grain size, cation distribution, density, and porosity^57^. Figures (8: a–h) illustrate the frequency-dependent dielectric and electrical properties of CoFe₂O₄ nanoparticles synthesized using (EG) and (PEG) methodologies over a frequency range from 1 Hz to 20 MHz. As shown in Fig. 8a, the real part of the dielectric permittivity ($\:{\epsilon\:}^{{\prime\:}}$) exhibits considerable dispersion at lower frequencies (1 Hz − 10³ Hz) for both synthesis methods. With increasing frequency, ε′ gradually declines and stabilizes within the MHz range. This behavior is primarily attributed to space-charge effects and Maxwell–Wagner polarization, which arise from interfaces, grain boundaries, and electrode influences. Notably, CoFe₂O₄ nanoparticles produced via the PEG method exhibit a higher ($\:{\epsilon\:}^{{\prime\:}}$) attributed to enhanced dipolar polarization, space-charge phenomena, and a stronger interfacial polarization. The imaginary permittivity (ε″), which indicates dielectric loss, is shown in Fig. 8b. Both nanoparticle samples demonstrate elevated losses at low frequencies, which decrease as frequency increases. The PEG-derived nanoparticles exhibit greater ($\:{\epsilon\:}^{{\prime\:}{\prime\:}}$) values compared to the EG-derived ones; this difference is due to increased energy dissipation from charge migration and interfacial polarization. At higher frequencies, both samples show minimal dielectric loss, indicating intrinsic polarizability. However, the PEG-derived sample displays more pronounced dielectric losses at low frequencies, likely resulting from intensified charge-carrier hopping between Fe²⁺ and Fe³⁺ ions. Briefly, low-frequency behavior is mainly influenced by electrode or interfacial polarization and space-charge dynamics, while high-frequency responses are governed by intrinsic polarizability. Differences between PEG and EG samples reflect disparities in microstructure and interface density due to their synthesis methods.

The real part of the impedance ($\:{Z}^{{\prime\:}}$) continuously decreases with increasing frequency, as shown in Fig. 8c. This trend confirms the semiconducting nature of these ferrites. The reduction in impedance at higher frequencies results from increased conductivity, as more charge carriers are promoted at elevated frequencies. At lower frequencies, EG nanoparticles exhibit higher ($\:{Z}^{{\prime\:}}$) values (indicating greater resistance) compared to PEG samples, suggesting larger grain-boundary resistance. In contrast, the imaginary part of the impedance ($\:{Z}^{{\prime\:}}{\prime\:}$), presented in Fig. 8d, displays a clear relaxation peak in the mid-frequency region for EG, indicating significant interfacial polarization and specific relaxation behavior. The PEG curve is flatter, suggesting enhanced charge transport and fewer barrier effects. Peaks in ($\:{Z}^{{\prime\:}}{\prime\:}$) correspond to relaxation processes defined by a time constant (τ = 1/2πf peak). The pronounced ($\:{Z}^{{\prime\:}}{\prime\:}$) peak for EG suggests a dominant relaxation process, possibly related to grain-boundary blocking or a single relaxation time. Conversely, the subdued ($\:{Z}^{{\prime\:}}{\prime\:}$) for PEG aligns with a broader range of relaxation times or stronger short-range conduction, reducing the impedance peak. Overall, the impedance spectra indicate that EG samples possess larger grain-boundary resistance and more pronounced relaxation, while PEG samples exhibit increased conductivity and weaker blocking at interfaces.

The electrical modulus formalism, advantageous for minimizing electrode polarization effects, has been employed in this analysis^57,58^. The electrical modulus is defined as follows:

$$\:{M}^{*}={M}^{{\prime\:}}+j{M}^{{\prime\:}{\prime\:}}=j\omega\:{C}_{o}{Z}^{*}=j\omega\:{C}_{o}({Z}^{{\prime\:}}-j{Z}^{{\prime\:}{\prime\:}})$$

where, $\:\omega\:$ represents the angular frequency, and the geometric capacitance is given by $\:{(C}_{o}={\epsilon\:}_{o}A/d)$ ($\:\mathrm{w}\mathrm{i}\mathrm{t}\mathrm{h}\:d$ being the sample thickness, $\:A$ the electrode area, and $\:{\epsilon\:}_{o}$ the permittivity of free space). The real modulus ($\:{M}^{{\prime\:}}$) is observed in Fig. 8e to be relatively small at low frequencies, dominated by long-range electrode and space-charge effects influencing the permittivity (ε). As frequency increases, the real modulus value rises, reflecting the attenuation of electrode polarization. Notably, EG nanoparticles exhibit higher modulus values, indicating shorter relaxation times, consistent with more confined dipolar and hopping dynamics observed in EG. The imaginary modulus ($\:{M}^{{\prime\:}{\prime\:}}$), presented in Fig. 8f, reveals distinct relaxation peaks for both nanoparticle types, with the EG sample displaying more pronounced and broader peaks across varying frequencies. This trend suggests the occurrence of multiple relaxation processes related to localized charge-carrier hopping, including electron hopping between Fe²⁺ and Fe³⁺ ions or polaron hopping involving transition-metal ions^59^. The variations in peak positions between the EG and PEG samples underscore differences in their microstructural properties and charge transport mechanisms. The pronounced peak observed in EG implies substantial contributions from these relaxation processes. In contrast, the broader and shifted peak in (PEG) suggests the presence of more rapid or distributed relaxation mechanisms.

As illustrated in Fig. 8g, the AC conductivity exhibits an increasing trend with frequency at room temperature, which can be attributed to electron hopping between Fe²⁺ and Fe³⁺ ions located within octahedral sites. Notably, the substantial increase in the real part of the conductivity ($\:{\sigma\:}^{{\prime\:}}$) observed at elevated frequencies indicates a predominance of short-range hopping conduction, wherein localized charge carriers respond to the applied electric field. The synthesis of CoFe₂O₄ nanoparticles utilizing PEG results in enhanced ($\:{\sigma\:}^{{\prime\:}}$) values, likely indicating a greater population of mobile charge carriers or improved connectivity between particles^59^. Conversely, samples synthesized via the EG route demonstrate higher impedance and greater grain-boundary blocking, leading to increased resistivity. The imaginary conductivity ($\:{\sigma\:}^{{\prime\:}{\prime\:}}$), as shown in Fig. 8h, exhibits relatively low magnitude values but shows significant fluctuations at high frequencies, primarily reflecting the influences of real conductivity. Samples prepared using the EG method are characterized by lower (ε′/ε″) ratios, elevated real impedance, and sharper features at low frequencies, all of which point to diminished conductivity. In contrast, PEG-derived samples exhibit enhanced low-frequency permittivity and AC conductivity, particularly at higher frequencies, which can be attributed to augmented interfacial polarization effects. The observed differences in microstructural characteristics suggest that PEG is more advantageous for applications that prioritize AC conductivity, while EG is better suited for contexts where low dielectric loss is critical.

### Photocatalytic degradation of rhodamine B (RhB)

**Fig. 9:**
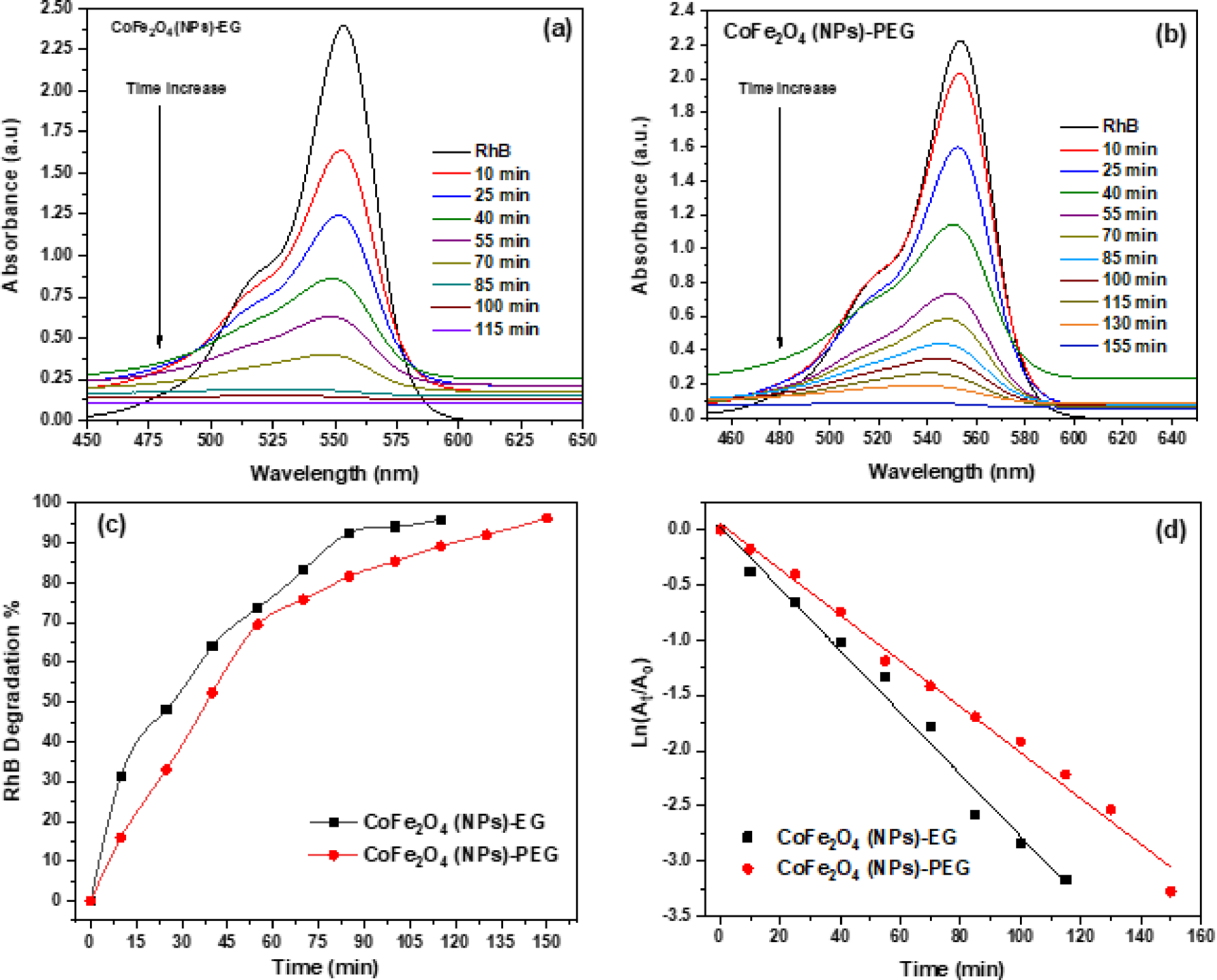
Description of the photocatalytic activity of CoFe₂O₄ nanoparticles capped with (EG) and (PEG) for Rhodamine B (RhB) degradation under light. (**a**,** b**) UV–Vis absorption spectra of RhB with CoFe₂O₄ (NPs)–EG and CoFe₂O₄ (NPs)–PEG. (**c**) RhB degradation efficiency over CoFe₂O₄ (NPs)–EG and CoFe₂O₄ (NPs)–PEG. (**d**) Pseudo-first-order kinetic plots of ln(C₀/C_t_) versus time.

The photocatalytic performance of cobalt ferrite nanoparticles (CoFe₂O₄), synthesized through (EG) and (PEG) methods, was systematically evaluated using Rhodamine B (RhB) as a model organic dye under UV irradiation (365 nm). Before illumination, the RhB-catalyst suspensions were magnetically stirred in the dark for 30 min to establish a state of adsorption-desorption equilibrium. Time-dependent UV-Vis spectral analysis (see Fig. 9: a, b) indicated a progressive attenuation of the characteristic RhB absorption band at approximately 554 nm, confirming the degradation of the dye over time^59,60^. The degradation efficiency was quantitatively assessed using the following equation:

$$\:Degradation\:\%=\frac{({A}_{o}-{A}_{t})}{{A}_{o}}\:\times\:100$$

where $\:{A}_{o}$ and $\:{A}_{t}$ represent the initial absorbance of the RhB dye and the absorbance values of RhB with CoFe₂O₄ nanoparticles after irradiation, respectively. Following 115 min of UV exposure, CoFe₂O₄ synthesized via the EG route achieved a degradation efficiency of approximately 95.8%. In contrast, the PEG-derived CoFe₂O₄ exhibited 89.1% degradation at the same time point, which increased to 96.2% after 150 min (see Fig. 9: c). These findings underscore the superior photocatalytic activity of CoFe₂O₄, particularly the EG-derived variant, in facilitating RhB degradation. Control experiments conducted without either light or catalyst demonstrated negligible removal of RhB, thereby confirming that the observed degradation process is predominantly governed by photocatalysis rather than through adsorption or photolysis mechanisms^61–63^. Kinetic analysis revealed that the degradation of RhB adhered to a pseudo-first-order model, as evidenced by the linear correlation between $\:Ln\:\left({A}_{t}/{A}_{o}\right)$ and irradiation time (see Fig. 9: d). The apparent rate constants were calculated to be 0.028 min⁻¹ for CoFe₂O₄–EG and 0.021 min⁻¹ for CoFe₂O₄–PEG, indicating rapid degradation kinetics when compared to previously reported ferrite-based photocatalysts.

**Fig. 10:**
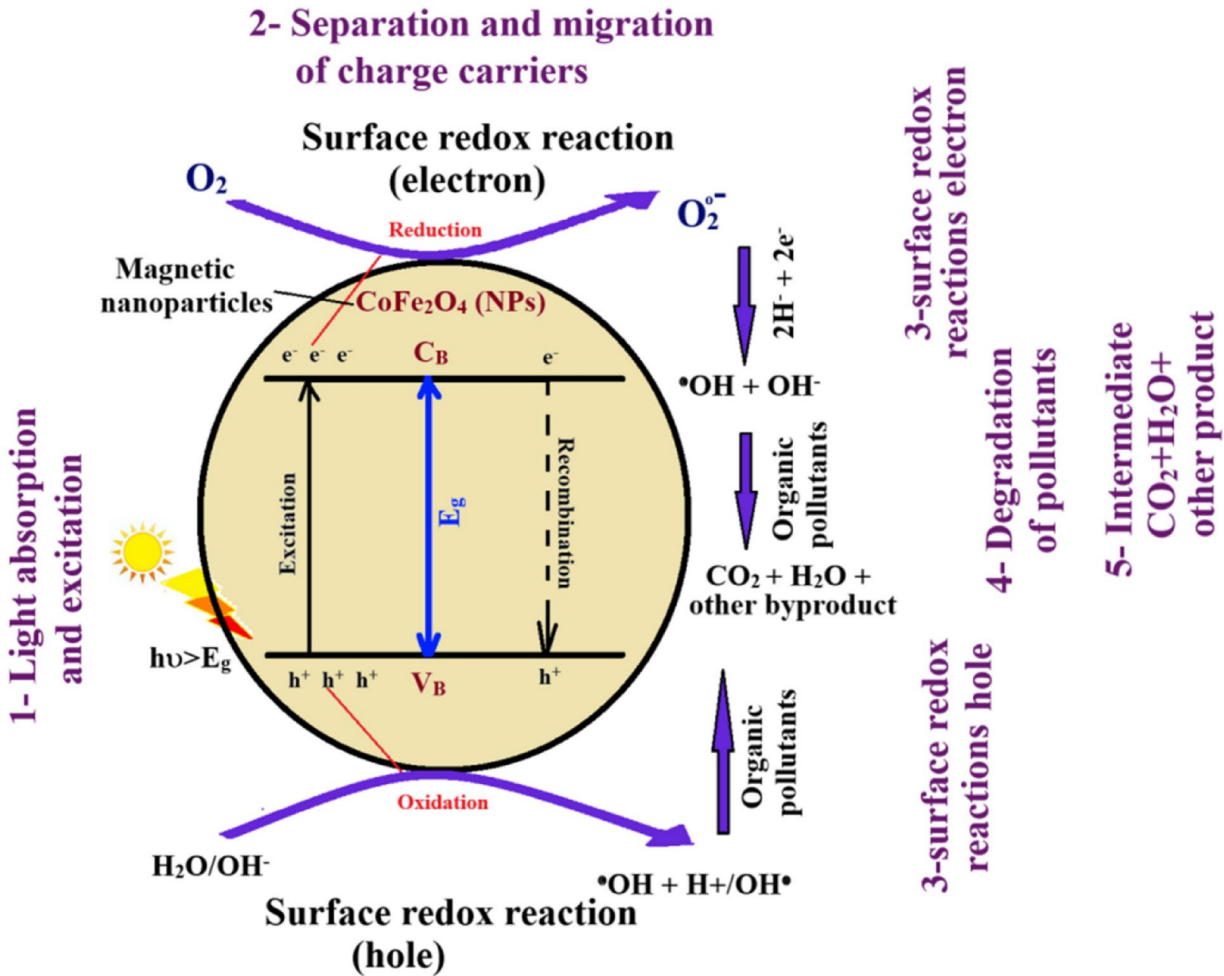
Description of the photocatalytic mechanism for pollutant degradation using CoFe₂O₄ nanoparticles. The process includes: (1) light absorption and excitation of electrons from the valence band (VB) to the conduction band (CB), (2) separation and migration of charge carriers, (3) surface redox reactions involving (**a**) electron-mediated reduction of O₂ to O₂⁻• and (**b**) hole-mediated oxidation of H₂O to •OH, (4) degradation of pollutants (P) into intermediates such as CO₂ and H₂O, and (5) magnetic recovery of the CoFe₂O₄ nanoparticles.

## Conclusion

This study explores how the choice of polyol, specifically (EG) versus (PEG), affects the properties of CoFe₂O₄ nanoparticles synthesized via the sol-gel method. While both types exhibited a cubic spinel structure, the PEG-capped nanoparticles showed superior crystallinity, larger sizes, and better dispersion. Optical analysis indicated narrow direct band gaps of 1.83 to 1.85 eV, ideal for visible light applications. The PEG-derived nanoparticles had a higher saturation magnetization of 70.1 emu·g⁻¹, whereas the EG samples displayed higher coercivity (547.9 Oe), highlighting a trade-off between the two. Dielectric measurements revealed improved permittivity and AC conductivity for the PEG nanoparticles. In photocatalytic tests for Rhodamine B degradation under UV light, the EG particles achieved 95.8% degradation in 115 min, while the PEG particles reached 96.2% in 150 min. These results suggest that selecting the appropriate polyol can help tailor CoFe₂O₄ nanoparticles for various applications, including magnetic storage, biomedical therapies, and wastewater remediation.

## Data Availability

The data supporting the findings of this study are available within the article. Additional data related to this study are available from the corresponding author upon reasonable request.

## References

[1] Pham, T. N., Huy, T. Q. & Le, A. T. Spinel ferrite (AFe2O4)-based heterostructured designs for lithium-ion battery, environmental monitoring, and biomedical applications. *RSC Adv.* **10** (52), 31622–31661. (2020). 10.1039/D0RA05133K 35520663 10.1039/d0ra05133k PMC9056412

[2] Bhosale, R. R., Shende, R. V. & Puszynski, J. A. Thermochemical water-splitting for H2 generation using sol-gel derived Mn-ferrite in a packed bed reactor. *Int. J. Hydrogen Energy*. **37** (3), 2924–2934. (2012). 10.1016/J.IJHYDENE.2011.03.010

[3] Salunkhe, A. B. et al. Polyvinyl alcohol functionalized cobalt ferrite nanoparticles for biomedical applications. *Appl. Surf. Sci. vol*. **264**, 598–604. (2013). 10.1016/J.APSUSC.2012.10.073

[4] Cao, Z. & Zuo, C. Direct synthesis of magnetic CoFe2O4 nanoparticles as recyclable photo-fenton catalysts for removing organic dyes. *ACS Omega*. **5** (35), 22614–22620. (2020). 10.1021/ACSOMEGA.0C03404 32923821 10.1021/acsomega.0c03404 PMC7482304

[5] Srinivasan, S. Y., Paknikar, K. M., Bodas, D. & Gajbhiye, V. Applications of cobalt ferrite nanoparticles in biomedical nanotechnology. *Nanomedicine* **13** (10), 1221–1238. (2018). 10.2217/NNM-2017-0379 29882719 10.2217/nnm-2017-0379

[6] Amiri, S. & Shokrollahi, H. The role of cobalt ferrite magnetic nanoparticles in medical science. *Mater. Sci. Eng. C*. **33** (1), 1–8. (2013). 10.1016/J.MSEC.2012.09.003 10.1016/j.msec.2012.09.003 25428034

[7] Burdett, J. K., Price, S. L. & Price, G. D. Role of the crystal-field theory in determining the structures of spinels. *J. Am. Chem. Soc.* **104** (1), 92–95. 10.1021/JA00365A019 (2002).

[8] Henderson, C. M. B., Charnock, J. M. & Plant, D. A. Cation occupancies in Mg, Co, Ni, Zn, Al ferrite spinels: a multi-element EXAFSstudy. *J. Phys. Condens. Matter*. **19** (7), 076214. (2007). 10.1088/0953-8984/19/7/076214 22251601 10.1088/0953-8984/19/7/076214

[9] Kombaiah, K. et al. Okra extract-assisted green synthesis of CoFe2O4 nanoparticles and their optical, magnetic, and antimicrobial properties. *Mater. Chem. Phys.* **204**, 410–419. (2018). 10.1016/J.MATCHEMPHYS.2017.10.077

[10] Zeng, X. L., Sivanesarajah, I. & Hartmann, U. Conversion of hard to soft magnetic ferrite nanowires by paramagnetic shielding. *Solids vol*. **4** (4), 304–315. (2023). 10.3390/SOLIDS4040019

[11] Kashid, P., Suresh, H. K., Mathad, S. N., Shedam, R. & Shedam, M. R. A review on synthesis, properties and applications on cobalt ferrite. *Int. J. Adv. Sci. Eng.* **9** (1), 2567. 10.29294/IJASE.9.1.2022.2567-2583 (2022).

[12] Cannas, C., Falqui, A., Musinu, A., Peddis, D. & Piccaluga, G. CoFe2O4 nanocrystalline powders prepared by citrate-gel methods: Synthesis, structure and magnetic properties. *J. Nanoparticle Res.* **8** (2), 255–267. (2006). 10.1007/S11051-005-9028-7

[13] Thomas, J. et al. Synthesis of cobalt ferrite nanoparticles by constant pH co-precipitation and their high catalytic activity in CO oxidation. *New. J. Chem. vol*. **41** (15), 7356–7363. (2017). 10.1039/C7NJ00558J

[14] Almeida, T. P., Fay, M., Zhu, Y. & Brown, P. D. Controlling role of pH and temperature on CoFe2O4 nanostructures produced by hydrothermal synthesis. *J. Nanosci. Nanotechnol vol*. **12** (11), 8801–8805. (2012). 10.1166/JNN.2012.6468 10.1166/jnn.2012.6468 23421290

[15] Duong, B. et al. Enhanced magnetism in highly ordered magnetite nanoparticle-filled nanohole arrays. *Small* **10** (no. (14)), pp2840–2848. (2014). 10.1002/SMLL.201303809 24706405 10.1002/smll.201303809

[16] Eom, Y., Abbas, M., Noh, H. Y. & Kim, C. G. Morphology-controlled synthesis of highly crystalline Fe3O4 and CoFe2O4 nanoparticles using a facile thermal decomposition method. *RSC Adv.* **6**, 15861–15867. (2016). 10.1039/C5RA27649G

[17] Dixit, C. K., Bhakta, S., Kumar, A., Suib, S. L. & Rusling, J. F. Fast nucleation for silica nanoparticle synthesis using a sol–gel method. *Nanoscale* **8** (47), 19662–19667. (2016). 10.1039/C6NR07568A 27858036 10.1039/c6nr07568a PMC5137187

[18] Singhal, A., Bisht, A., Kumar, A. & Sharma, S. One pot, rapid synthesis of Co3O4 by solution combustion method and its electrochemical properties in different electrolytes. *J. Electroanal. Chem.* **776**, 152–161. (2016). 10.1016/J.JELECHEM.2016.07.004

[19] Chen, W. et al. Manganese-enhanced MRI of rat brain based on slow cerebral delivery of manganese(II) with silica-encapsulated MnxFe1-xO nanoparticles. NMR Biomed. **26** (9) 1176–1185 (2013). 10.1002/nbm.2932 23526743 10.1002/nbm.2932

[20] Boni, A., Albertazzi, L., Innocenti, C., Gemmi, M. & Bifone, A. Water dispersal and functionalization of hydrophobic iron oxide nanoparticles with lipid-modified Poly(amidoamine) dendrimers. *Langmuir* **29** (35), 10973–10979. (2013). 10.1021/LA400791A 23721318 10.1021/la400791a

[21] Majewski, A. P. et al. PDMAEMA-grafted core-shell-corona particles for nonviral gene delivery and magnetic cell separation. *Biomacromolecules vol*. **14** (9), 3081–3090. (2013). 10.1021/BM400703D 10.1021/bm400703d 23889326

[22] Ruhland, T. M., Lang, J. R. V., Alt, H. G. & Müller, A. H. E. Magnetic core-shell nanoparticles as carriers for olefin dimerization catalysts. *Eur. J. Inorg. Chem.* (2013). 10.1002/EJIC.201201547

[23] Zimny, K. et al. Design of a fluorinated magneto-responsive material with tuneable ultrasound scattering properties. *J. Mater. Chem. B*. **2** (10), 1285–1297. (2014). 10.1039/C3TB21585G 32261443 10.1039/c3tb21585g

[24] Chang, Y., Yang, C., Zheng, X. Y., Wang, D. Y. & Yang, Z. G. Fabrication of copper patterns on flexible substrate by patterning–adsorption–plating process. *ACS Appl. Mater. Interfaces*. **6** (2), 768–772. (2014) 10.1021/AM405539R 24386981 10.1021/am405539r

[25] Malinowska, I., Ryżyńska, Z., Mrotek, E., Klimczuk, T. & Zielińska-Jurek, A. Synthesis of CoFe2O4 nanoparticles: the effect of ionic strength, concentration, and precursor type on morphology and magnetic properties. *J. Nanomater*. (2020). 10.1155/2020/9046219

[26] Fouad, D. E., Zhang, C., El-Didamony, H., Yingnan, L. & Mekuria, T. D. Ahmer Hussain Shah, Improved size, morphology and crystallinity of hematite (α-Fe2O3) nanoparticles synthesized via the precipitation route using ferric sulfate precursor. *Results Phys.* **12**, 1253–1261. 10.1016/j.rinp.2019.01.005 (2019).

[27] Hubbard, C. R. & Snyder, R. L. RIR - Measurement and Use in Quantitative XRD. *Powder Diffr.* **3** (2), 74–77. 10.1017/S0885715600013257 (1988).

[28] Bowles, J. F. W. & Cornell, R. M. and U. Schwertmann the iron oxides: structure, properties reactions occurrence and uses. Weinheim and New York (VCH Verlagsgeseiischaft mbH). 1996, xxxi + 573 pp. Price DM 328.00. ISBN 3-527-28576-8., Mineral. Mag., **61 **(408), 740–741, 1997). 10.1180/MINMAG.1997.061.408.20

[29] Liu, C., Rondinone, A. J. & Zhang, Z. J. Synthesis of magnetic spinel ferrite CoFe2O4 nanoparticles from ferric salt and characterization of the size-dependent superparamagnetic properties, Pure Appl. 72 (1–2), 37–45, (2000). 10.1351/PAC200072010037/XML

[30] Köseoǧlu, Y. et al. Synthesis and characterization of ZnFe2O4 magnetic nanoparticles via a PEG-assisted route, J. Alloys Compd. **462** (1–2) 209–213, (2008). 10.1016/J.JALLCOM.2007.07.121

[31] Langford, J. I. & Wilson, A. J. C. Scherrer after sixty years: A survey and some new results in the determination of crystallite size, J. Appl. Crystallogr. **11** (2) 102–113,(1978). 10.1107/S0021889878012844

[32] Makuła, P., Pacia, M. & Macyk, W. How To Correctly Determine the Band Gap Energy of Modified Semiconductor Photocatalysts Based on UV-Vis Spectra, J. Phys. Chem. Lett. **9** (23) 6814–6817, (2018). 10.1021/ACS.JPCLETT.8B02892/SUPPL_FILE/JZ8B02892_LIVESLIDES.MP4 10.1021/acs.jpclett.8b02892 30990726

[33] Hoang, V. H. et al. One-Step hydrothermal synthesis and characterization of highly dispersed Sb-doped SnO2 nanoparticles for supercapacitor applications. *Electrochem* **6** (2), 22. 10.3390/ELECTROCHEM6020022/S1 (2025).

[34] Pegah & Baminejhad Enayatollah Sheikhhosseini,Mahdieh Yahazadehfar, Synthesis of cobalt- ferrite and zinc oxide metal nanoparticles based-bentonite using SDS and their investigation as catalysts in synthesis of benzylbarbiturocoumarins, ORIGINAL RESEARCH article 12–2024 | 10.3389/fchem.2024.1434488 10.3389/fchem.2024.1434488 PMC11345271 39189017

[35] El-Ghazzawy, E. H., Saafan, S. A., Abo-aita, N. M. & Eid, M. A. Atlam,Enhanced physical properties and antimicrobial activity of PEG-4000 capping on CoxCu1-xFe2O4 nanoparticles for technological applications: Synthesis and characterization. *J. Solgel Sci. Technol.* **116**, 1797–1820. 10.1007/s10971-025-06974-4 (2025).

[36] Habibi, M. H. & Parhizkar, H. J. FTIR and UV–vis diffuse reflectance spectroscopy studies of the wet chemical (WC) route synthesized nano-structure CoFe2O4 from CoCl2 and FeCl3, Spectrochim. Acta Part A Mol. Biomol. Spectrosc., vol. 127, pp. 102–106 (2014). 10.1016/J.SAA.2014.02.090 10.1016/j.saa.2014.02.090 24637270

[37] Chakhtouna, H. et al. Functional CoFe2O4-modified biochar derived from banana pseudostem as an efficient adsorbent for the removal of amoxicillin from water, Sep. Purif. Technol., vol. 266. (2021). 10.1016/j.seppur.2021.118592

[38] Thomas, A., Müller, S. S. & Frey, H. Beyond poly(ethylene glycol): Linear polyglycerol as a multifunctional polyether for biomedical and pharmaceutical applications. *Biomacromolecules* **15** (6), 1935–1954. 10.1021/BM5002608 (2014). 10.1021/bm5002608 24813747

[39] Stern, T., Deciphering the triple-peak, C-O-C. & stretching FTIR absorbance consistently occurring in semicrystalline PEG. *Polym.*10.3390/POLYM17162199 (2025). 10.3390/polym17162199 PMC12389251 40871149

[40] Abdelsalam, H. K. et al. Magnetoelastic correlation in annealed cobalt-bismuth nanoferrites: Linking vibrationlastic, and magnetic properties for high-frequency soft-magnetic applications, Ceramics International, 52(15) 29010–29018, 10.1016/j.ceramint.2026.04.429. (2026).

[41] Gopal, M. Y. Physico-chemical characterization of cobalt ferrite nano-particles synthesized via sol-gel method,Educ. Adm. *Theory Pract.* (2024). 10.53555/KUEY.V30I3.9839

[42] Mary Jacintha, A., Umapathy, V., Neeraja, P. & Rajkumar, S. R. J. Synthesis and comparative studies of MnFe2O4 nanoparticles with different natural polymers by sol–gel method: structural, morphological, optical, magnetic, catalytic and biological activities. *J. Nanostructure Chem.* **7** (4), 375–387. (2017). 10.1007/S40097-017-0248-Z

[43] Hunpratub, S., Phokha, S., Kidkhunthod, P., Chanlek, N. & Chindaprasirt, P. The effect of cation distribution on the magnetic properties of CoFe2O4 nanoparticles. *Results Phys.* (2021). 10.1016/J.RINP.2021.104112

[44] Farshad Farshidfar, A., Fattahi, R., Brüning, Dominic, H., Ryan & Ghandi, K. A creative method to tune Fe–O interaction in ferrites. *J. Adv. Ceram.* **12**, 1612–1624. 10.26599/JAC.2023.9220775 (2023). https://www.sciopen.com/article/

[45] Chen, J., Wu, X. & Selloni, A. Electronic structure and bonding properties of cobalt oxide in the spinel structure. *Phys. Rev. B*. **83**, 245204. 10.1103/PhysRevB.83.245204 (2011).

[46] Bandigowdanahalli Prabhuswamy, V. et al. Enhanced optical and UV shielding performance of CoFe2O4@GONDs embedded PVA nanocomposites, Polymer (Guildf). 339, 129137, (2025). 10.1016/J.POLYMER.2025.129137

[47] Inbaraj, D. J., Chandran, B. & Mangalaraj, C. Synthesis of CoFe2O4 and CoFe2O4/g-C3N4 nanocomposite via honey mediated sol-gel auto combustion method and hydrothermal method with enhanced photocatalytic and efficient Pb + 2 adsorption property. *Mater. Res. Express*. **6** (5), 055501. 10.1088/2053-1591/AAFD5D (2019).

[48] Murphy, A. B. Optical properties of an optically rough coating from inversion of diffuse reflectance measurements. *Appl. Opt.* **46** (16), 3133–3143. 10.1364/AO.46.003133 (2007). 17514266 10.1364/ao.46.003133

[49] Tatarchuk, T. et al. Structural, Optical, and Magnetic Properties of Zn-Doped CoFe2O4 Nanoparticles. *Nanoscale Res. Lett.* **12** (1), 141. 10.1186/s11671-017-1899-x (2017). 28235377 10.1186/s11671-017-1899-x PMC5319947

[50] Abul Kalam, A. G. et al. Modified solvothermal synthesis of cobalt ferrite (CoFe_2_O_4_) magnetic nanoparticles photocatalysts for degradation of methylene blue with H2O2/visible light, Results in Physics, **8**, Pages 1046–1053, ISSN 2211–3797, (2018). 10.1016/j.rinp.2018.01.045

[51] Vlazan, P. & Stoia, M. Structural and magnetic properties of CoFe2O4 nanopowders, prepared using a modified Pechini method. *Ceram. Int.* **44** (1), 530–536. 10.1016/J.CERAMINT.2017.09.207 (2018).

[52] Omelyanchik, A. et al. Magnetocrystalline and surface anisotropy in CoFe2O4 nanoparticles. *Nanomater* **10** (7), 1288. (2020). 10.3390/NANO10071288 10.3390/nano10071288 PMC7408426 32629977

[53] Chinnasamy, C. N. et al. Growth dominant co-precipitation process to achieve high coercivity at room temperature in CoFe2O4 nanoparticles,* IEEE Trans. Magn.* **38**, 5 2640–2642 10.1109/TMAG.2002.801972 (2002).

[54] Abdelsalam, H. K., Asmaa, A. H., Part, A. & El-Bassuony & Tuning structural, magnetic, and antimicrobial properties of novel nanocomposites to be applied in biomedical applications. *Ceram. Int.* **51** (30), 63138–63150 (2025). 10.1016/j.ceramint.2025.11.088. (2025).

[55] Abdelsalam, H. K., Asmaa, A. H., Part, A. & El-Bassuony & Novel equal-ratio Nanocomposites: Attractive physical properties for high-frequency and antimicrobial biomedical applications. *Ceram. Int.* **51** (25), 44506–44519. 10.1016/j.ceramint.2025.07.180 (2025).

[56] Abdelsalam, H. K. & El-Bassuony, A. A. H. Study of spinel-based nanocomposites. *Eur. Phys. J. Spec. Top.*10.1140/epjs/s11734-026-02157-6 (2026).

[57] Hoffmann, M. R., Martin, S. T., Choi, W. & Bahnemann, D. W. Environmental Applications of Semiconductor Photocatalysis. *Chem. Rev.* **95** (1), 69–96. 10.1021/CR00033A004 (2002).

[58] Mahdikhah, V., Saadatkia, S., Sheibani, S. & Ataie, A. Outstanding photocatalytic activity of CoFe2O4 /rGO nanocomposite in degradation of organic dyes. *Opt. Mater. (Amst)*. 10.1016/J.OPTMAT.2020.110193 (2020).

[59] Naik, M. M. et al. Photocatalytic degradation of dyes by cobalt ferrite nanoparticles synthesized by sol-gel method, AIP Conf. Proc., 2274, 1, (2020). 10.1063/5.0022559/1026259

[60] Sahmi, A., Bensadok, K. & Trari, M. Electrochemical properties of CoFe2O4 prepared by sol–gel route. Sono-photocatalysis degradation of Rhodamine B by solar light. *React. Kinet Mech. Catal.* **137** (3), 1823–1837. (2024). 10.1007/S11144-024-02608-Y

[61] Butler, M. A. & Ginley, D. S. Prediction of Flatband Potentials at Semiconductor-Electrolyte Interfaces from Atomic Electronegativities. *J. Electrochem. Soc.* **125** (2), 228. (1978). 10.1149/1.2131419

[62] Yong, X. & Schoonen, M. A. A. The absolute energy positions of conduction and valence bands of selected semiconducting minerals. *Am. Mineral.* **85** (3–4), 543–556. (2000). 10.2138/AM-2000-0416

[63] Pavel, M. et al. Photocatalytic degradation of organic and inorganic pollutants to harmless end products: assessment of practical application potential for water and air cleaning. *Catal* **13** (2), 380. (2023). 10.3390/CATAL13020380

[64] Alharthi, F. A. & Hasan, I. Cobalt oxide–copper oxide heterojunction nanocomposites for efficient UV-driven photocatalytic degradation of Brilliant Blue G dye. *J. Sol-Gel Sci. Technol.* **117** (3), 86–1173. (2026). 10.1007/S10971-026-07137-9

[65] Lei, P. et al. Degradation of dye pollutants by immobilized polyoxometalate with H 2O2 under visible-light irradiation. *Environ. Sci. Technol.* **39** (21), 8466–8474. (2005). 10.1021/ES050321G 16294889 10.1021/es050321g

[66] Zhang, H., Chen, G. & Bahnemann, D. W. Photoelectrocatalytic materials for environmental applications. *J. Mater. Chem.* **19** (29), 5089–5121. (2009). 10.1039/B821991E

